# Supplementary material for: Gut microbiota regulates acute myeloid leukaemia via alteration of intestinal barrier function mediated by butyrate
Source: Nat Commun. 2022 May 9;13:2522. doi: 10.1038/s41467-022-30240-8 (PMC9085760; doi:10.1038/s41467-022-30240-8)
Supplement: Supplementary file 1 — Supplementary Information [file 41467_2022_30240_MOESM1_ESM.pdf]

Supplementary Information File

Gut microbiota regulates acute myeloid leukaemia via alteration of intestinal barrier function mediated by butyrate

Ruiqing Wang[1], Xinyu Yang[1], Jinting Liu[1], Fang Zhong[2], Chen Zhang[1], Yuhong Chen[1], Tao Sun[1], Chunyan Ji[1], Daoxin Ma[1]*

[1]Department of Hematology, Qilu Hospital of Shandong University, Jinan, Shandong 250012, P.R. China; [2]Department of Endocrinology and Metabolism, Shandong Provincial Hospital affiliated with Shandong University, Jinan, Shandong 250012, P.R. China

Corresponding author: Daoxin Ma, Professor

Address: Department of Hematology, Qilu Hospital of Shandong University, Jinan, Shandong 250012, P.R. China

Phone: 86-0531-82169887 Fax: 86-0531-86927544

E-mail address: daoxinma@sdu.edu.cn

This file includes:

Supplementary Figures 1 to 11

Supplementary Tables 1 to 3

Supplementary figure 1

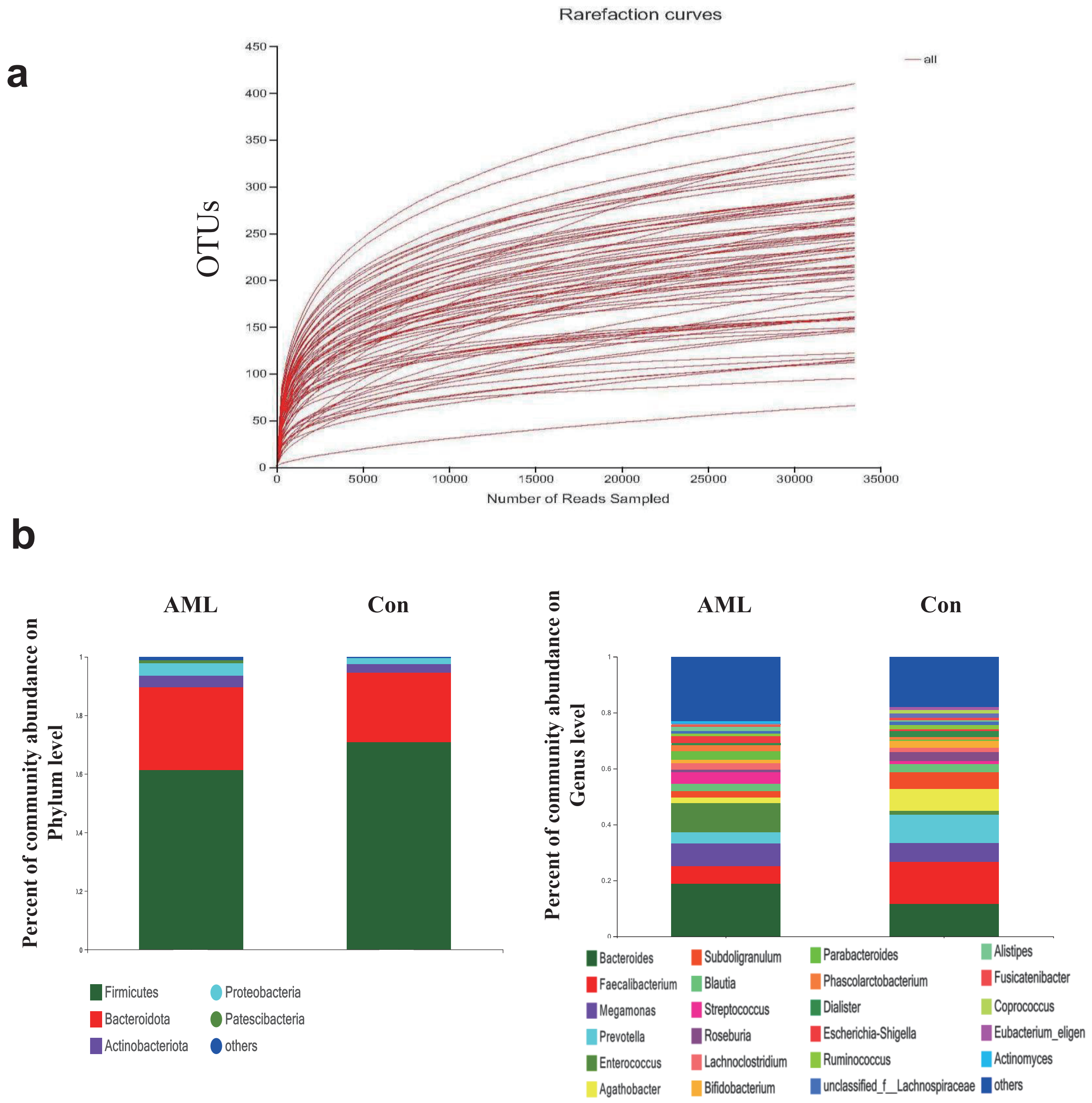

Supplementary figure 1

a Rarefaction curves of 61 samples from AML patients and healthy controls.

b The difference in abundances of phylum and genus between the AML and control groups.

Supplementary figure 2

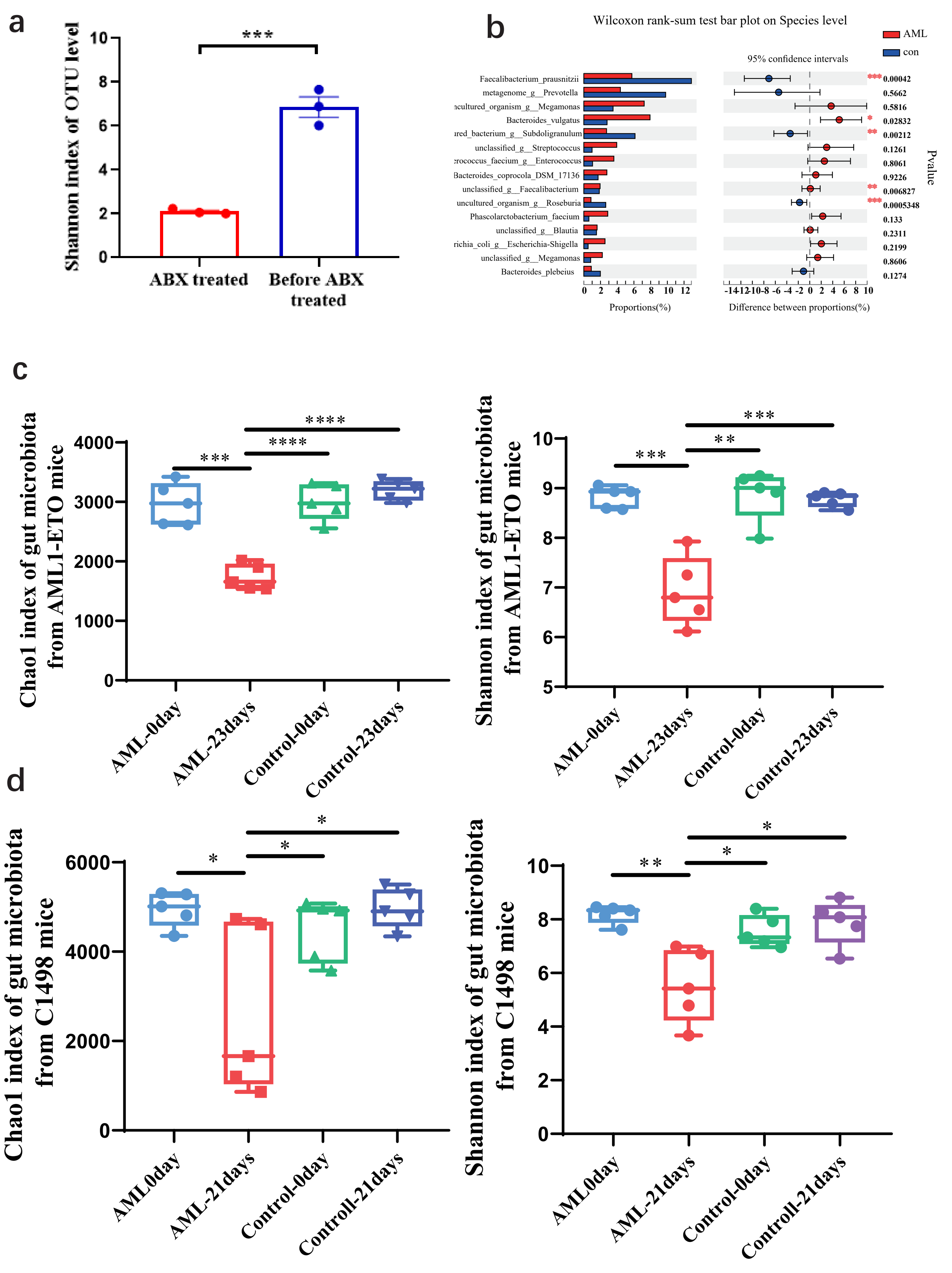

e

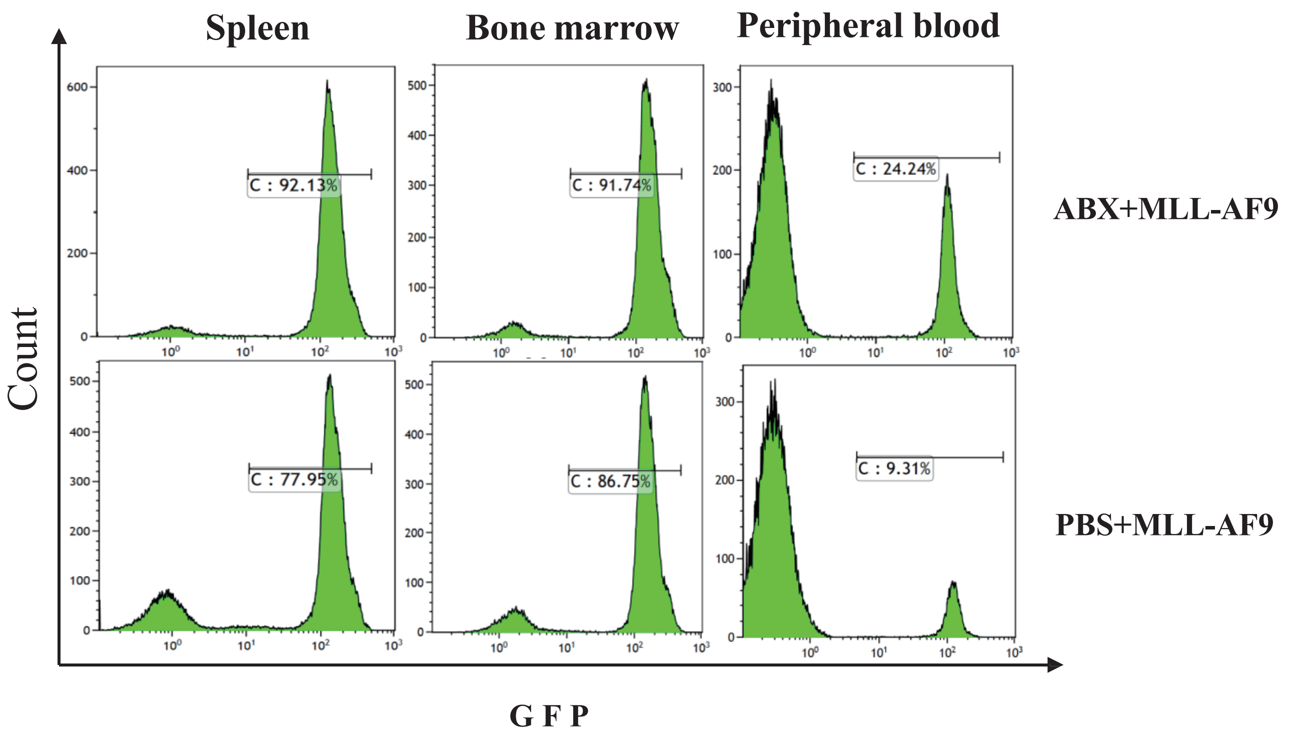

Spleen
Bone marrow
Peripheral blood
Count
C : 92.13%
C : 91.74%
C : 24.24%
ABX+MLL-AF9
C : 77.95%
C : 86.75%
C : 9.31%
PBS+MLL-AF9
G F P

Supplementary figure 2

a The diversity and abundance of the gut microbiota in ABX-treated and control mice. Unpaired t-test was used for comparing the Sob index (n=3 per group). **b** Relat ive OTUS taxon abundance comparison among the AML (n=31) and control groups (n=30). The mean is used to measure the centre of the error bar. **c** Chaol and shannon index of gut microbiota of AMLl-ETO mice (n=5 per group). **d** Chaol and shannon index of gut microbiota of C1498 mice (n=5 per group). **c-d** Data were presented as standard boxplots (with the box encompassing Q1 to Q3, the median denoted as a central horizontaline in the box, and the whiskers covering the data within ±1.5 IQR). **e** The representative FACS graphs of the leaukemia cells (GFP+ cells) in spleen, peripheral blood and bone marrow from ABX AML mice and control PBS AML mice. P-values were determined using Two- tailed t-test in **a, c, d** and Wilcoxon rank test in **b**. Error bars represent mean ± SEM in **a-d**. ***p=0.0006 **(a)**, ***p=0.0004 AML-23 days vs. AML-0 day shannon, **p=0.001 Control-0 day vs. AML-23 days shannon, ***p=0.0004 Control-23 days vs. AML-23 days shannon, ***p=0.0002 AML-23 days vs. AML-0 day chao1, ****p<0.0001 Control-0 day vs. AML-23 days chao1, ****p<0.0001 Control-23 days vs. AML-23 days chao1 **(c)**, **p=0.003 AML-21 days vs. AML-0 day shannon, *p=0.0157 Control-0 day vs. AML-21 days shannon, *p=0.0113 Control-23 days vs. AML-21 days shannon, *p=0.00273 AML-21 days vs. AML-0 day chao1, *p=0.0157 Control-0 day vs. AML-21 days chao1, *p=0.0113 Control-21 days vs. AML-21 days chao1 **(d)**. Source data are provided as a Source Data file.

Supplementary figure 3

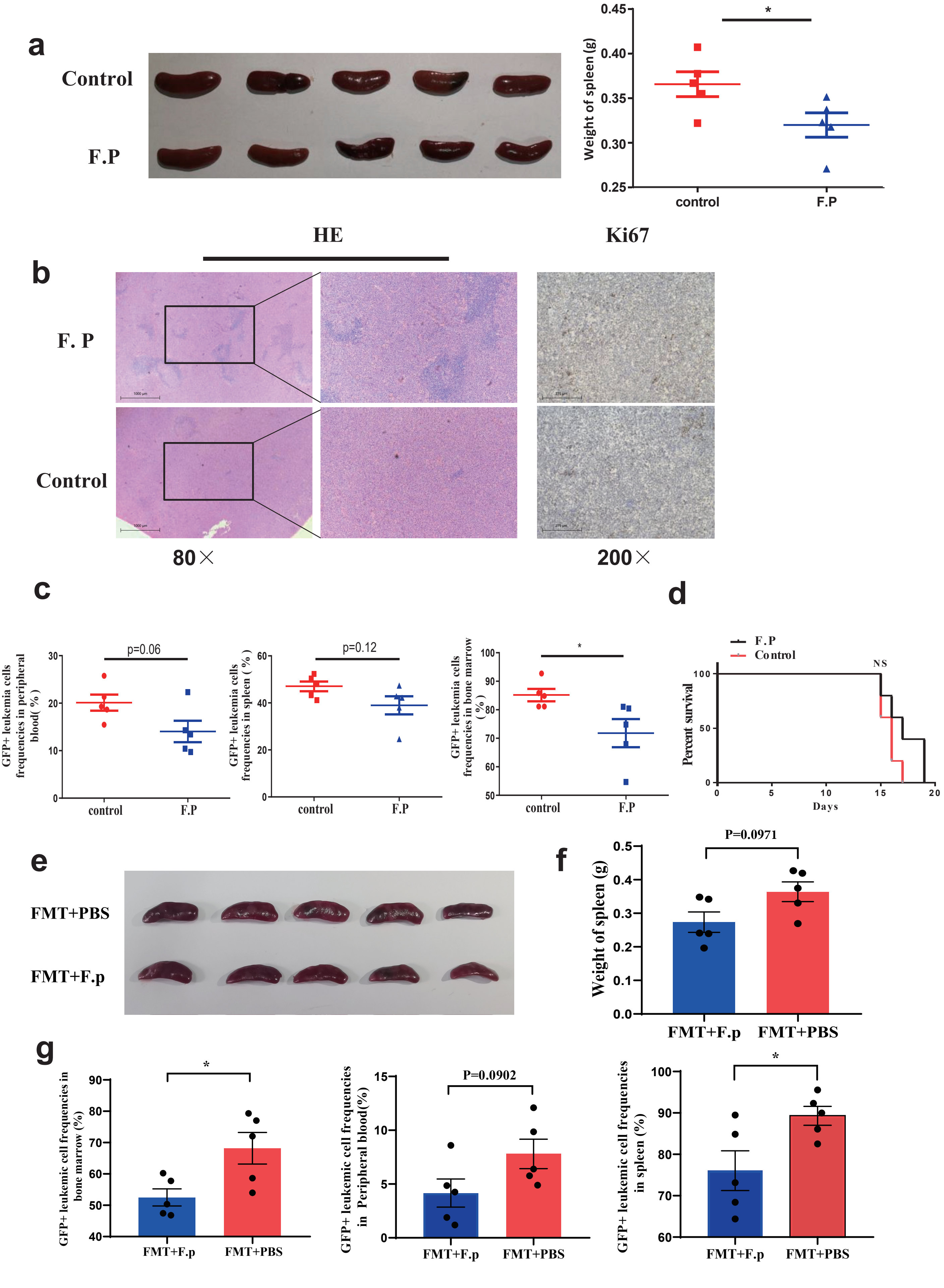

Supplementary figure 3

a Photograph of spleens from FP treated AML mice (n=5) and control AML mice (n=5). **b** HE histopathology sections and Ki67 immunohistochemical staining of a representative spleen, F.P treated group and control group. All microscopic analyses were performed (original magnification x80 or x200), scale bar =1000μm and 275um. **c** The cells (GFP+ cells) in the spleen, peripheral blood and bone marrow from F.P treated AML mice (n=5) and control AML mice (n=5). **d** Kaplan- Meier survival curve of AML mice (n=5 per group). **e** Photograph of spleens from AML mice treated with FP or without F.P (n=5 per group). **f** Weight of spleens from AML mice treated with F.P or without F.P (n=5 per group). P-values were determined using Two-tailed t-test in **a, c, f, g**. P-values were determined using Gehan-Breslow-Wilcoxon test in **d**. Error bars represent mean ± SEM. *p=0.0468**(a)**, *p=0.0366 BM **(c)**, *p=0.0258 SP, *p=0.0376 BM **(g)**. Source data are provided as a Source Data file.

Supplementary figure 4

a

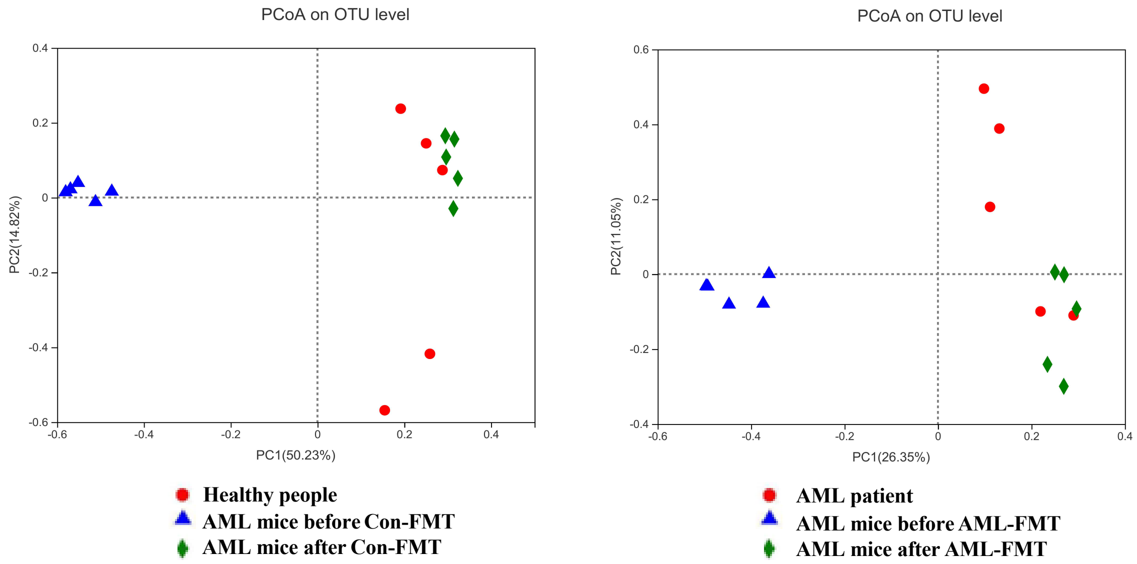

b

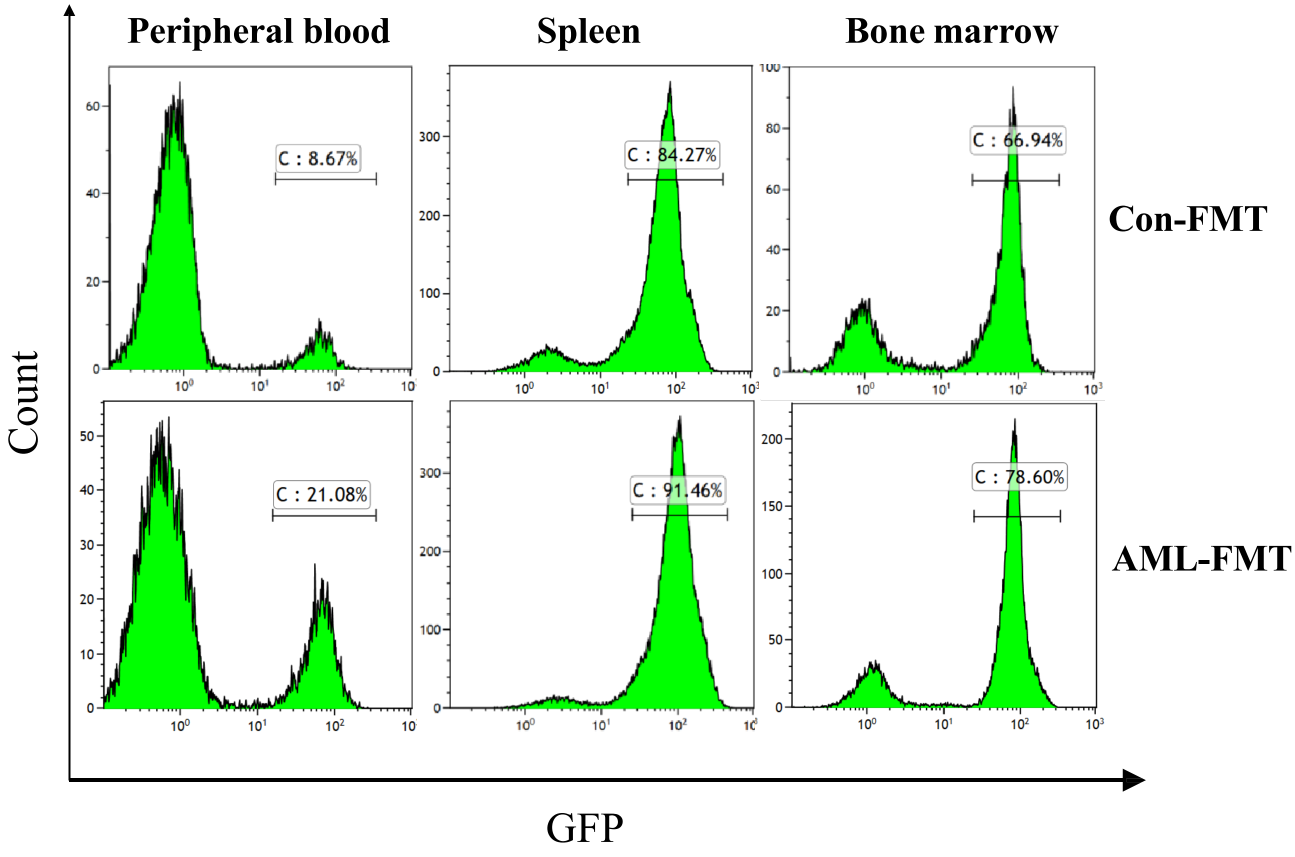

Supplementary figure 4

a PCoA of a Weighted UniFrac distance analysis. **b** The representative FACS graphs of the leukaemia cells (GFP+ cells) in spleen, peripheral blood and bone marrow.

Supplementary figure 5

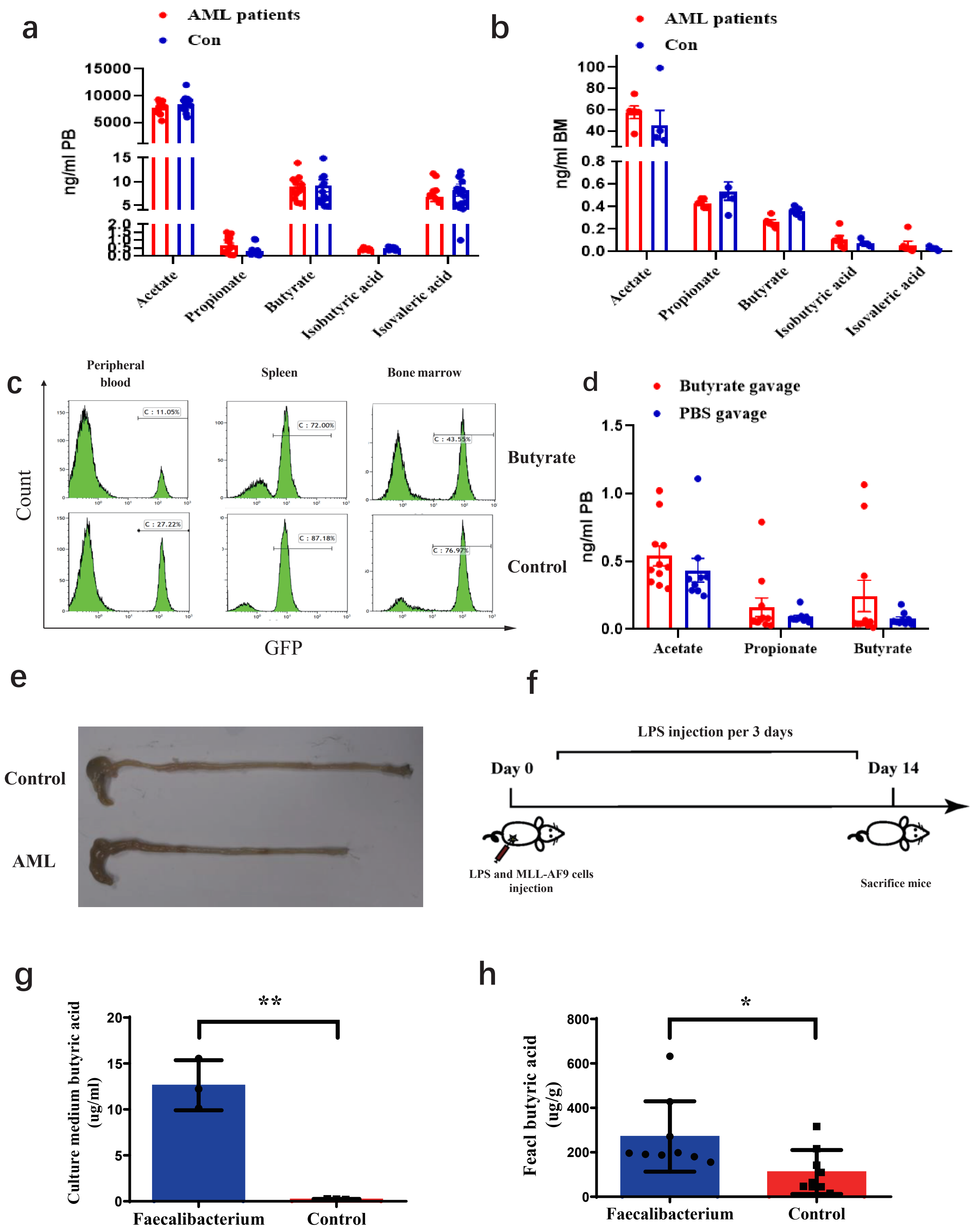

Supplementary figure 5

a The concentrations of SCFAs in peripheral blood of AML patients (n=20) and controls (n=22). **b** The concentrations of SCFAs in bone marrow of AML patients (n=7) and controls (n=5). **c** The representative FACS graphs of the leukemia cells (GFP+ cells) in the spleen, peripheral blood and bone marrow. **d** The concentrations of SCFAs in blood samples of butyrate treatment mice (n=11) and controls (n=9). **e** The colon length of AML mice and control mice. **f** The schematic diagram of the mice experimental process including LPS treatment. **g** The concentration of butyrate in the supernatant of Faecalibacterium (n=3 per group). **h** The butyrate content in mice gavaged with Faecalibacterium compared with controls (n=9, per group). **g-h** All data are from the independent experiments. P-values were determined using Two-tailed t-test in **a, b, d, g, h**. Error bars represent mean ± SEM. **p=0.0014 **(g)**, *p=0.0207 **(h)**. Source data are provided as a Source Data file.

Supplementary figure 6

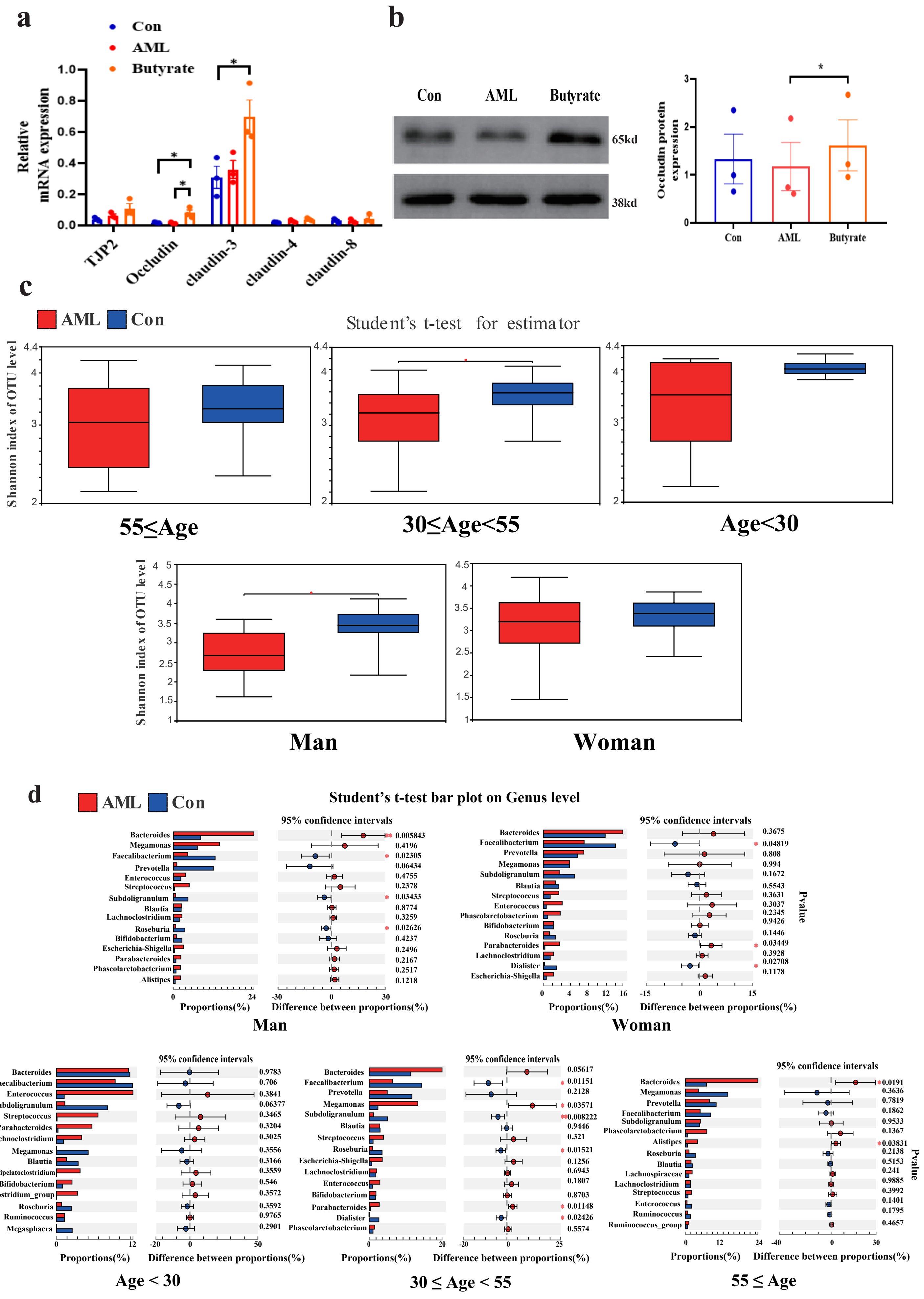

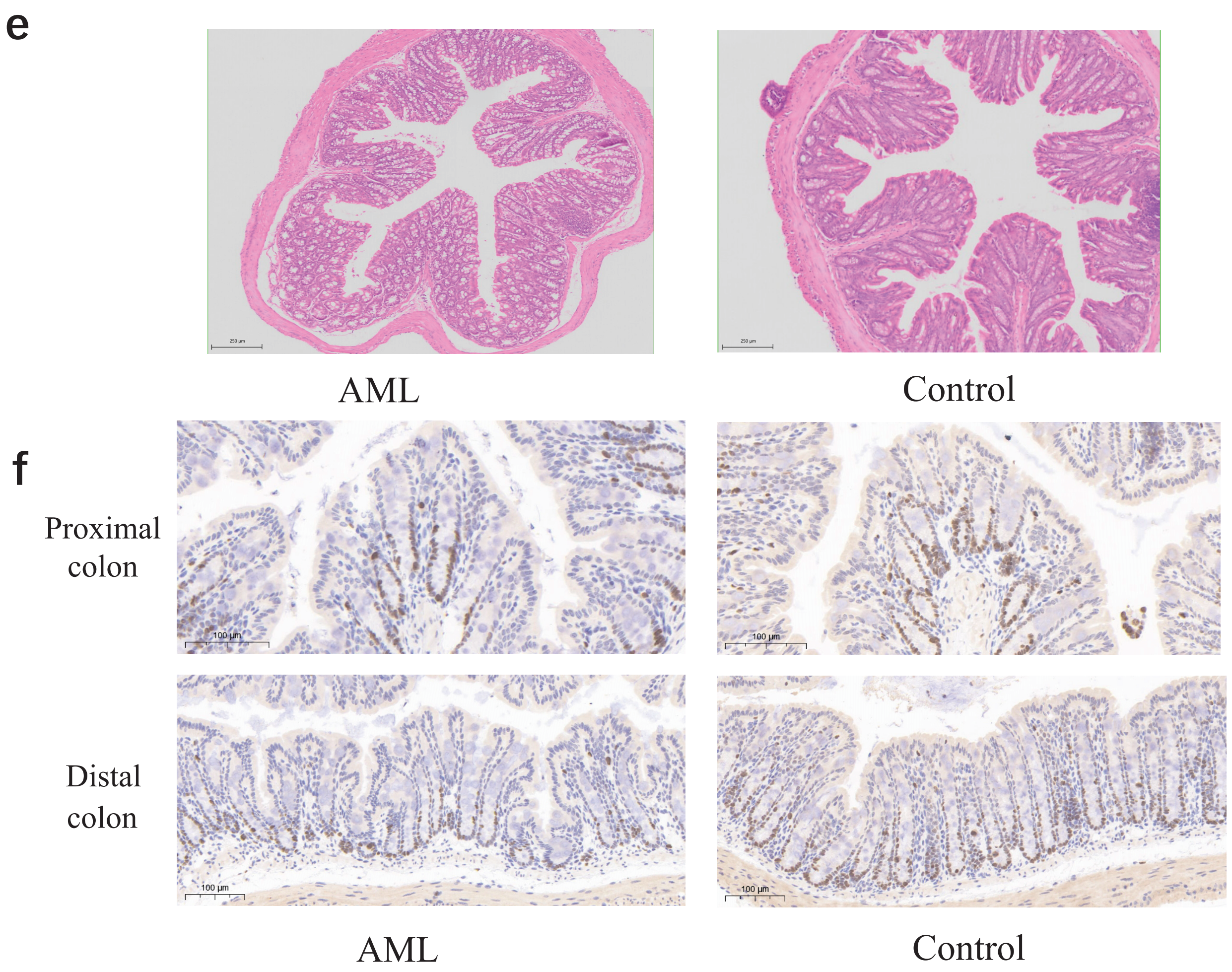
e
AML
Control
f
Proximal colon
Distal colon
100 µm
AML
Control

Supplementary figure 6

a The mRNA expression levels of tight junction protein components TJP-2, occludin, claudin-3, claudin-4 and claudin-8 in intestinal epithelial cells of AML, control and butyrate-treatment mice (n=3 per group). **b** The protein levels of occluding in intestinal epithelial cells were determined by Western blot. GAPDH was used as the control (n=3, per group). **c** Diversity and richness of the gut microbiota in AML patients (AML) and healthy controls (Con) (differentiated by age and gender). The unpaired t test was used to compare the Shannon index (n=61). Data were presented as standard boxplots (with the box encompassing Q1 to Q3, the median denoted as a central horizontal line in the box, and the whiskers covering the data within ±1.5 IQR). **d** Relative taxa abundance comparison among the AML and control groups (differentiated by age and gender) (n=61). The mean is used to measure the centre of the error bar. **e-f** The colon HE and Ki67 staining of AML and control mice, scale bar=250μm and 100μm. P-values were determined using Two-tailed t-test and error bars represent mean ± SEM in **a-i**. *p=0.0373 AML vs AML+ Butyrate (Occludin), *p=0.0323 AML+ Butyrate vs Control (Occludin), *p=0.0416 AML vs AML+ Butyrate (claudin-3) **(a)**. *p=0.0109 AML+Butyrate vs Control (Occludin) **(b)**. *p=0.01225 30<Age<55 group, *p=0.01822 Man group **(c)**. Source data are provided as a Source Data file.

Supplementary figure 7

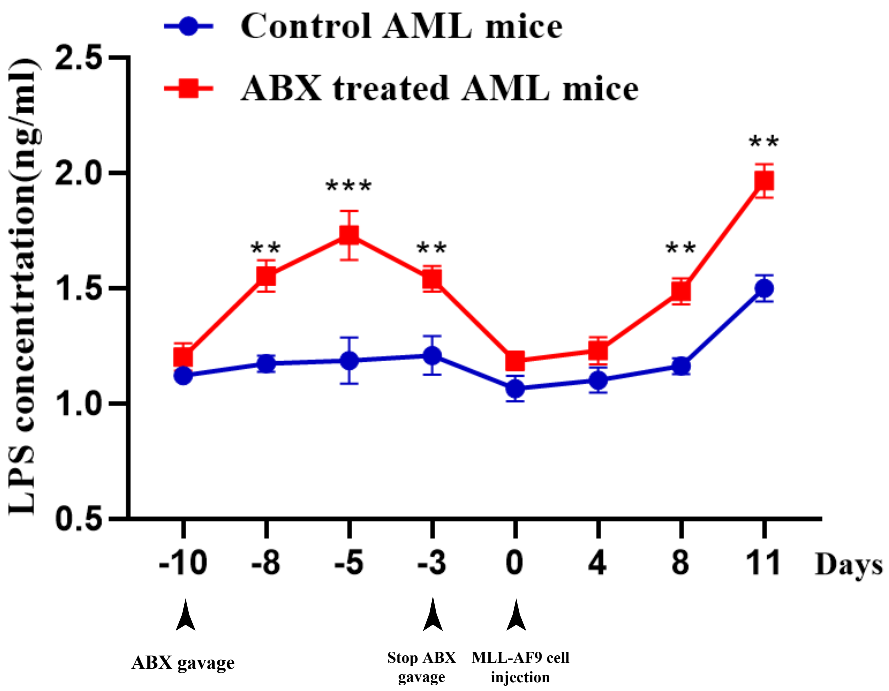

Supplementary figure 7

LPS concentrations in the peripheral plasma of ABX treated AML mice and control AML mice (-10-11 days)(n=4 per group). P-values were determined using Two-tailed t-test and Error bars represent mean ± SEM. **p=0.002543 -8days, **p=0.009798 -5days, *p=0.016498 -3days, *p=0.010607 8days, **p=0.004464 11days. Source data are provided as a Source Data file.

Supplementary figure 8

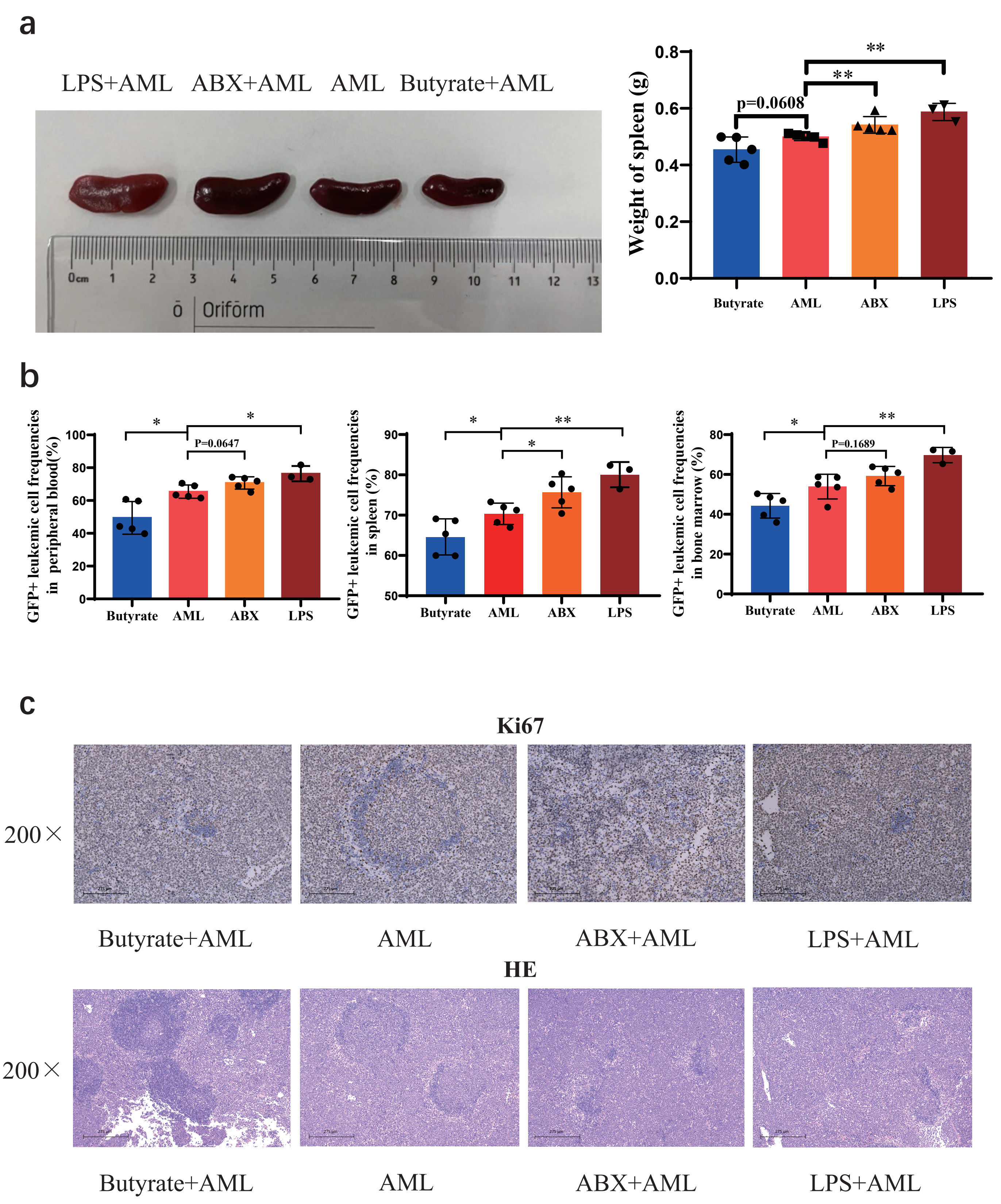

Supplementary figure 8

a Photographs and weights of spleens from AML mice(n=5), butyrate treated AML mice (n=5), ABX treated AML mice (n=5) and LPS treated AML mice (n=3). **b** Leukaemia cells (GFP^+ cells) in the spleen, peripheral blood, and bone marrow from AML mice (n=5), butyrate treated AML mice (n=5), ABX treated AML mice (n=5) and LPS treated AML mice (n=3). **c** Ki67 immunohistochemical and HE histopathology sections staining of a representative splee n, AML mice, butyrate treated AML mice, ABX treated AML mice and LPS treated AML mice, scale bar = 275 µm. P-values were determined using Two-tailed t-test and error bars represent mean ± SEM in **a, b**. **p=0.006265 AML vs ABX , **p=0.00117 AML vs LPS **(a)**, *p=0.0105 AML vs Butyrate+AML PB, *p=0.0126 AML vs LPS PB, *p=0.0388 AML vs Butyrate+AML SP, *p=0.0338 AML vs ABX SP, **p=0.00328 AML vs LPS SP, *p=0.0389 AML vs Butyrate+AML BM, **p=0.0076 AML vs LPS BM **(b)**. Source data are provided as a Source Data file.

Supplementary figure 9

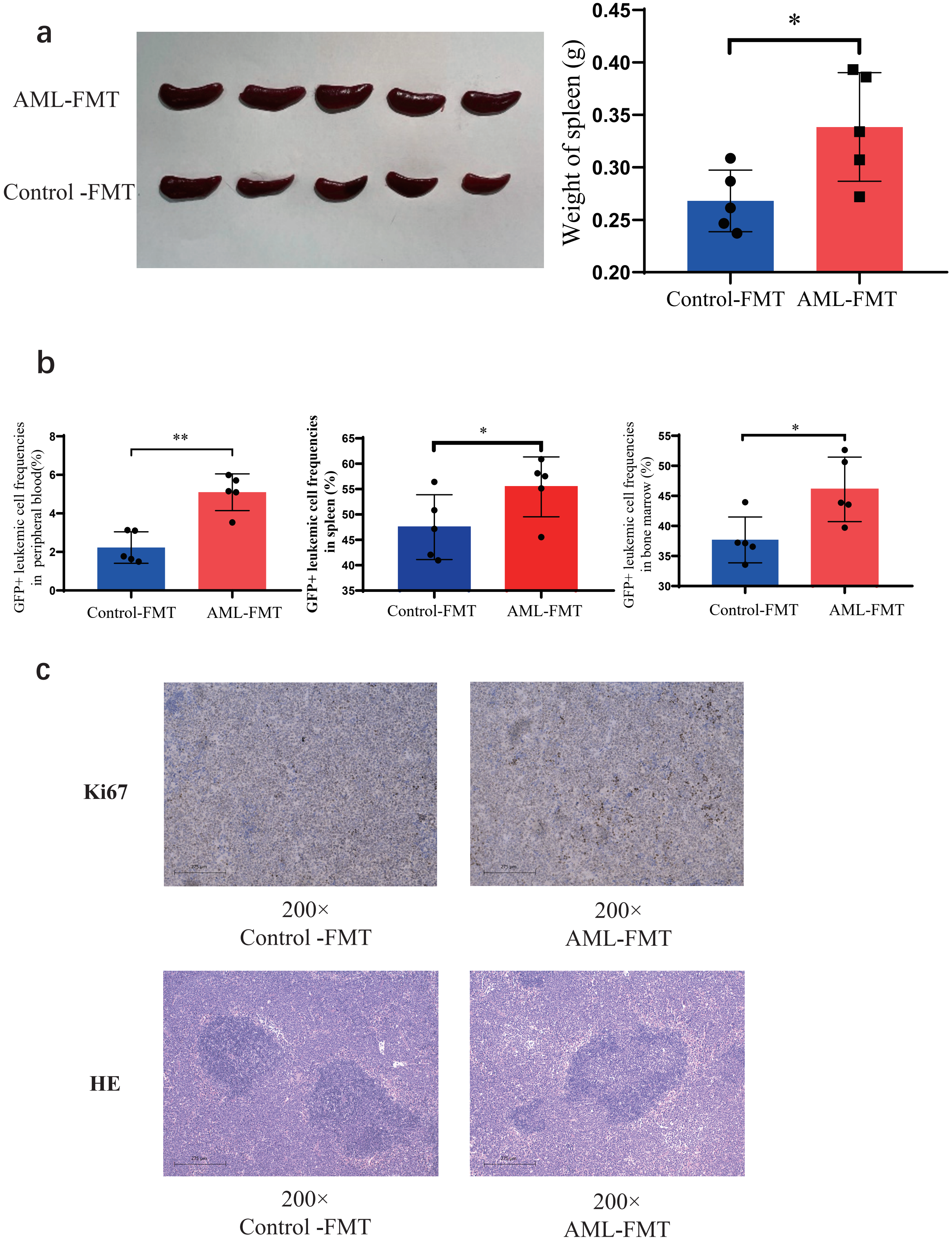

Supplementary figure 9

a Photographs and weights of spleens from AML-FMT group (n=5) and Control-FMT group (n=5). **b** Leukaemia cells (GFP+ cells) in the spleen, peripheral blood, and bone marrow from AML- FMT group (n=5) and Control-FMT group (n=5). P-values were determined using Two-tailed t-test and error bars represent mean ± SEM in **a, b**. **c** HE histopathology sections and Ki67 immunohistochemical staining of a representative spleen, AML- FMT group and Control-FMT group, scale bar=275 μm. *p=0.292 **(a)**, ***p=0.0009 PB, *P=0.0472 SP, *p=0.0214 BM **(b)**. Source data are provided as a Source Data file.

Supplementary figure 10

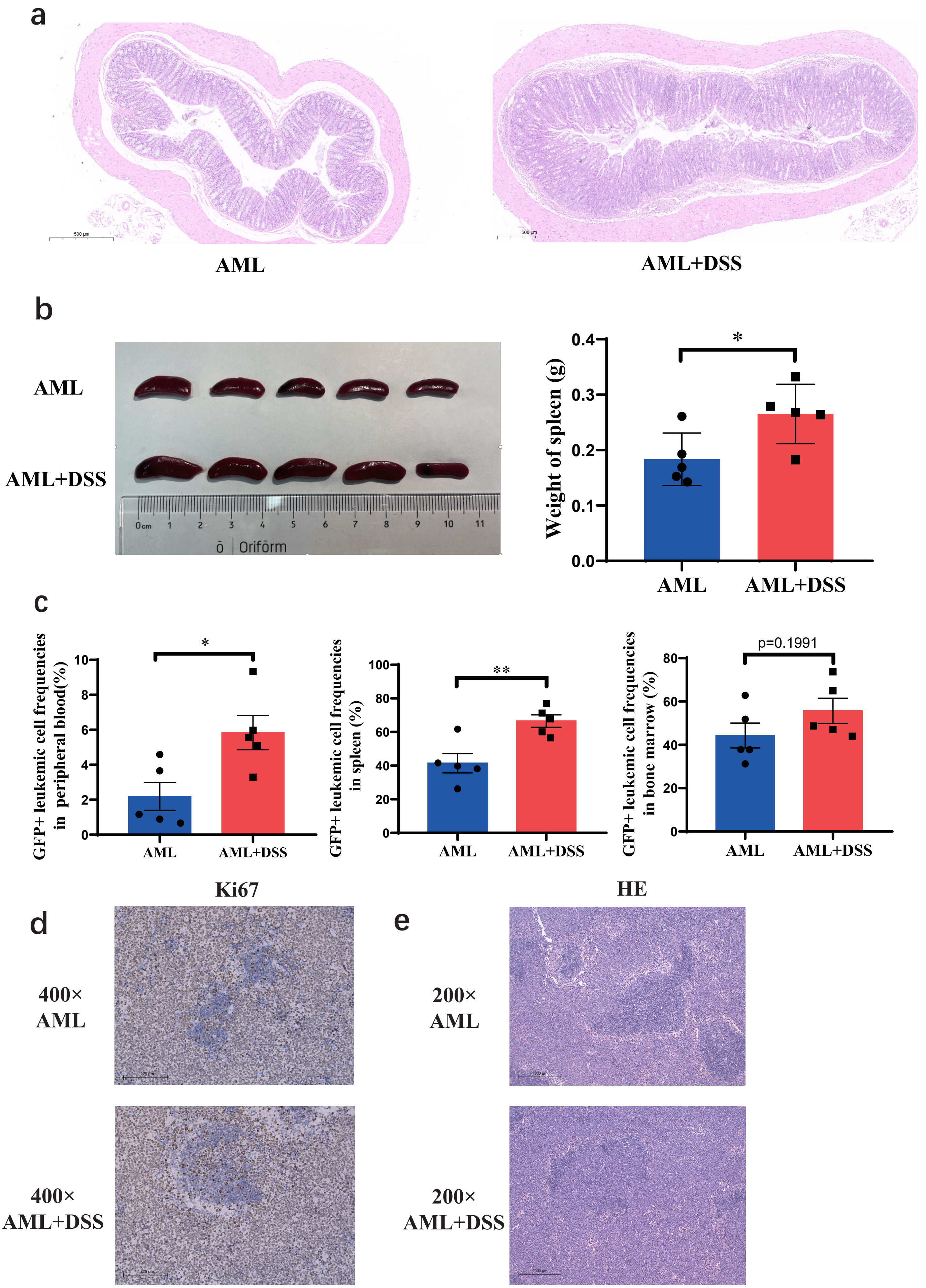

Supplementary figure 10

a The colon HE staining of AML and DSS treated AML mice. **b** Photographs and weights of spleens from AML (n=5) and DSS treated AML mice (n=5), scale bar = 500μm. Three times each experiment was repeated independently with similar results. **c** Leukaemia cells (GFP^+ cells) in the spleen, peripheral blood, and bone marrow from AML (n=5) and DSS treated AML mice (n=5). **d** Ki67 immunohistochemical staining of a representative spleen from DSS-treated AML mice, and control AML mice. **e** HE histopathology sections staining of a representative spleen, DSS-treated AML mice, and control AML mice, scale bar=275 μm. P-values were determined using Two-tailed t-test and error bars represent mean ± SEM in **b**, **c**. *p=0.034 **(b)**, *p=0.0205, **p=0.0063 **(c)**. Source data are provided as a Source Data file.

Supplementary figure 11

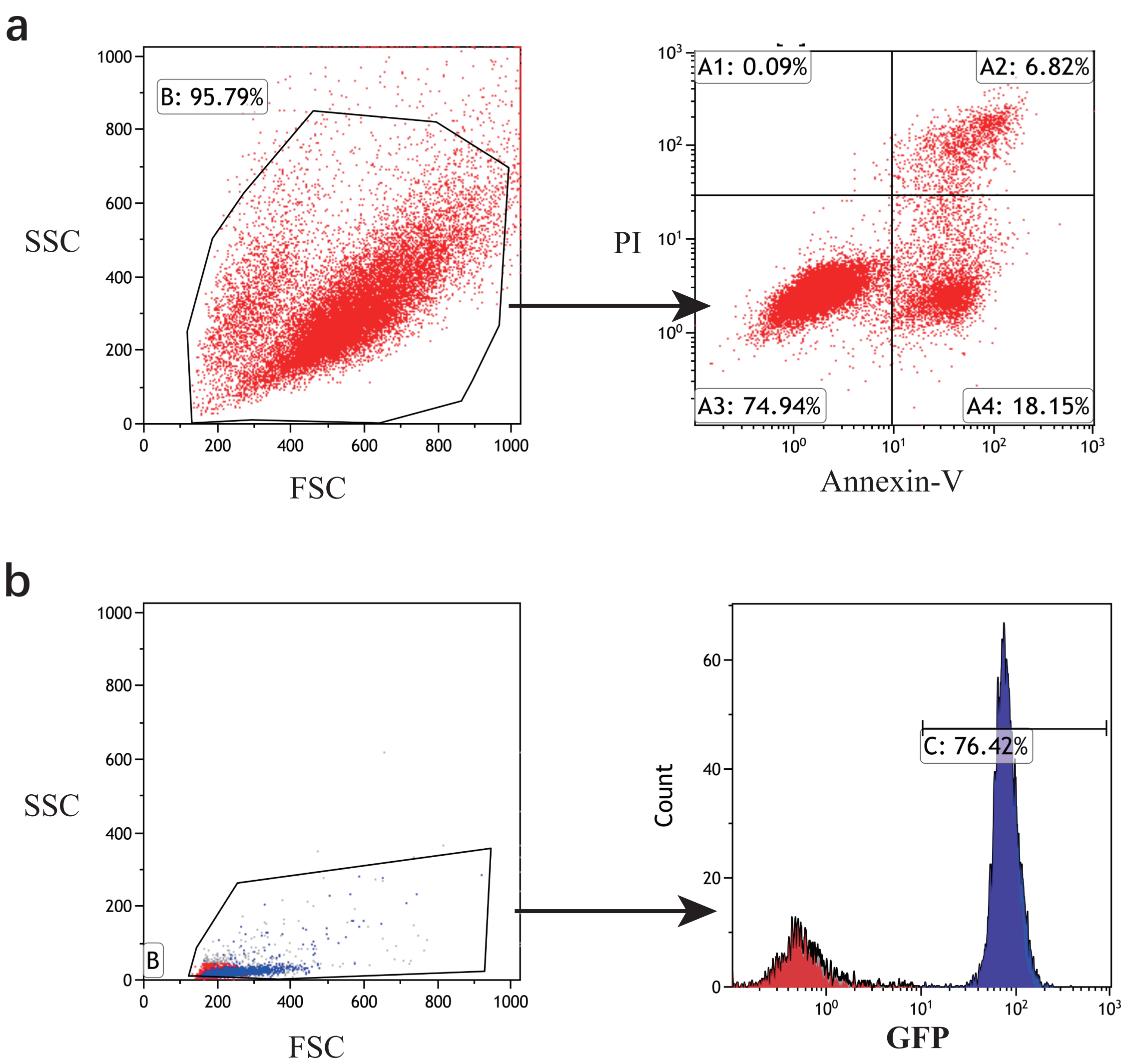

Supplementary figure 11

a Gating strategy was used to identify apoptotic cells in mouse primary cells. Annexin-V was used to label early apoptotic cells, and Annexin-V and PI were used to label late apoptotic cells. **b** Gating strategy was used to identify GFP^{+} cells in mouse peripheral blood, spleen and bone marrow cells.

Supplementary table 1. Clinical and laboratory characteristics of the 31 AML cases and 30 controls.

Characteristics	AML Patient	Healthy control
Male/female	15/16	15/15
Age at study entry, year, median (range)	46（15-69）	49（20-73）
Bone marrow blasts at diagnosis, %, median (range)	73（26-96）	/
WBC at diagnosis, $\times10^9$/L, median (interquartile range)	31.7（0.82-467）	/
Cytogenetic risk group, no./total no.		
Favorable	9/31	/
Unfavorable	20/31	/
Missing data	2/31	/
Status after the first course of chemotherapy, no./total no. (%)		
CR	20/31	/
Not CR	9/31	/
Unavailable data	2/31	/
AML FAB subtype, no./total no. (%)		
AML with minimal differentiation: M0	0/31	/
AML without maturation: M1	1/31	/
AML with maturation: M2	2/31	/
Acute Promyelocytic leukemia: M3	7/31	/
Acute Myelomonocytic leukemia: M4	3/31	/
Acute monoblastic or monocytic leukemia: M5	14/31	/
Acute erythroid leukemia: M6	0/31	/
Acute megakaryoblastic leukemia: M7	0/31	/
Other subtype	4/31	/

WBC: white blood cell; CR: complete remission

Supplementary table 2

The sequences of PCR primers

Gene	Primers (5' - 3')	
	**Forward**	**Reverse**
mClaudin-1	AGGTCTGGCGACATTAGTGG	TGGTGTTGGGTAAGAGGTTG
mClaudin-2	CTCCCTGGCCTGCATTATCTC	ACCTGCTACCGCCACTCTGT
mZO-1	GCCGCTAAGAGCACAGCAA	TCCCCACTCTGAAAATGAGGA
mTJP2	ATGGGAGCAGTACACCGTGA	TGACCACCCTGTCATTTTCTTG
mOccludin	TTGAAAGTCCACCTCCTTACAGA	CCGGATAAAAAGAGTACGCTGG
mClaudin-3	ACCAACTGCGTACAAGACGAG	CAGAGCCGCCAACAGGAAA
mClaudin-4	TGGAGGACGAGACCGTCAA	CACGGGCACCATAATCAGCA
mClaudin-8	GCAACCTACGCTCTTCAAATGG	TTCCCAGCGGTTCTCAAACAC
mGAPDH	GGACACTGAGCAAGAGAGGC	TTATGGGGGTCTGGGATGGA

Risk classification standard

Risk Category	Genetic Abnormality
**Favorable-risk**	t (8;21) (q22;q22.1);RUNX1-RUNX1T1<br>inv(16)(p13.1q22) or t(16;16)(p13.1;q22);CBFB-MYH11Biallelic mutated CEBPA<br>Mutated NPM1 without FLT3-ITD or with FLT3-ITD^{low}
**Unfavorable-risk (Intermediate and Adverse)**	Mutated NPM1 and FLT3-ITD^{high}<br>Wild-type NPM1 without FLT3-ITD or with FLT3-ITD^{low} (without adverse-risk genetic lesions) t(9;11)(p21.3;q23.3);MLLT3-KMT2A<br>Cytogenetic abnormalities not classified as favorable or adverse<br>t(6;9)(p23;q34.1);DEK-NUP214t(v;11q23.3);KMT2A rearranged t(9;22)(q34.1;q11.2);BCR-ABL1<br>inv(3)(q21.3q26.2) or t(3;3)(q21.3;q26.2);GATA2,MECOM(EV/1)-5 or del(5q);-7;-17/abn(17p)<br>Complex karyotype, monosomal karyotype‖ Wild-type NPM1 and FLT3-ITD^{high}<br>Mutated RUNX1, Mutated ASXL1, Mutated TP53

Reference：Acute Myeloid Leukemia, Version 3.2019, NCCN Clinical Practice Guidelines in Oncology

Supplementary table 3

Multiple linear regression analysis

Variables	Parameter Estimates		
	B	P	Exp (B)
Faecalibacterium	-30.055	0.002	-0.640
Roseburia	-3.518	0.638	-0.087
Age	-0.394	0.713	-0.055

Dependent Variable: WBC; B: regression coefficient; Exp(B): The exponent of B； Italic: Bacterial species name

The risk classification standard is listed in supplementary Table 2

A two-tailed P-value < 0.05 was considered statistically significant

Risk-prognosis assessment

	favorable	unfavorable	Total
CR	9	11	20
Non-CR	0	9	9
Total	9	20	29

Patients' information

	Gender	Positive genes
Patient-1	Male	FLT3-ITD, NPM1, IDH2,
Patient-2	Male	SRSF2 CEBPA
Patient-3	Male	FLT3-ITD, NPM1, DNMT3A
Patient-4	Female	CBFB-MYH11
Patient-5	Female	IDH2, NPM1
