# Supplementary material for: Gut microbiota regulates acute myeloid leukaemia via alteration of intestinal barrier function mediated by butyrate
Source: Nat Commun. 2022 May 9;13:2522. doi: 10.1038/s41467-022-30240-8 (PMC9085760; doi:10.1038/s41467-022-30240-8)
Supplement: Supplementary file 3 — Reporting Summary [file 41467_2022_30240_MOESM3_ESM.pdf]

nature research

Corresponding author(s): Daoxin Ma

Last updated by author(s): Mar 19, 2022

Reporting Summary

Nature Research wishes to improve the reproducibility of the work that we publish. This form provides structure for consistency and transparency in reporting. For further information on Nature Research policies, see our Editorial Policies and the Editorial Policy Checklist.

Statistics

For all statistical analyses, confirm that the following items are present in the figure legend, table legend, main text, or Methods section.

n/a	Confirmed	
[ ]	[x]	The exact sample size (*n*) for each experimental group/condition, given as a discrete number and unit of measurement
[ ]	[x]	A statement on whether measurements were taken from distinct samples or whether the same sample was measured repeatedly
[ ]	[x]	The statistical test(s) used AND whether they are one- or two-sided<br>*Only common tests should be described solely by name; describe more complex techniques in the Methods section.*
[x]	[ ]	A description of all covariates tested
[x]	[ ]	A description of any assumptions or corrections, such as tests of normality and adjustment for multiple comparisons
[ ]	[x]	A full description of the statistical parameters including central tendency (e.g. means) or other basic estimates (e.g. regression coefficient) AND variation (e.g. standard deviation) or associated estimates of uncertainty (e.g. confidence intervals)
[ ]	[x]	For null hypothesis testing, the test statistic (e.g. *F*, *t*, *r*) with confidence intervals, effect sizes, degrees of freedom and *P* value noted<br>*Give P values as exact values whenever suitable.*
[x]	[ ]	For Bayesian analysis, information on the choice of priors and Markov chain Monte Carlo settings
[x]	[ ]	For hierarchical and complex designs, identification of the appropriate level for tests and full reporting of outcomes
[ ]	[x]	Estimates of effect sizes (e.g. Cohen's *d*, Pearson's *r*), indicating how they were calculated

Our web collection on statistics for biologists contains articles on many of the points above.

Software and code

Policy information about availability of computer code

Data collection	No software was used
Data analysis	GraphPad Prism 8.0, SPSS 20.0, ImageJ V1.50, Kaluzza 2.1.1,AMT 700 camera

For manuscripts utilizing custom algorithms or software that are central to the research but not yet described in published literature, software must be made available to editors and reviewers. We strongly encourage code deposition in a community repository (e.g. GitHub). See the Nature Research guidelines for submitting code & software for further information.

Data

Policy information about availability of data

All manuscripts must include a data availability statement. This statement should provide the following information, where applicable:

- Accession codes, unique identifiers, or web links for publicly available datasets
- A list of figures that have associated raw data
- A description of any restrictions on data availability

Some or all data, models, or code generated or used during the study are available from the corresponding author by request. Our sequencing data were compared with KEGG and COG databases for further analysis. The metabolic raw datasets generated in this study have been deposited in the metabolights database under accession code MTBLS3092 [http://www.ebi.ac.uk/metabolights/editor/study/MTBLS3104/files] and 16s rRNA sequencing datasets were uploaded to the NCBI database with SRA, accession: PRJNA746137 [http://www.ncbi.nlm.nih.gov/sra/?term=PRJNA746137].

Field-specific reporting

Please select the one below that is the best fit for your research. If you are not sure, read the appropriate sections before making your selection.

- [x] Life sciences
- [ ] Behavioural & social sciences
- [ ] Ecological, evolutionary & environmental sciences

For a reference copy of the document with all sections, see nature.com/documents/nr-reporting-summary-flat.pdf

Life sciences study design

All studies must disclose on these points even when the disclosure is negative.

Sample size	Sample sizes were chosen based on preliminary data demonstrating statistically significant differences for each specific assay.
Data exclusions	The exclusion criteria were as follows: age <15 years and > 70 years, diarrhea, receiving antibiotics or hormone therapy within the last 10 weeks, and blood pressure anomalies.
Replication	All experiments were successfully performed with at least three technical replicates on more than one occasion to ensure reproducibility across experiments.
Randomization	The mice used in the experiments were randomly divided into different treatment groups. We also randomly selected one subset from the participants (patients or controls) meeting the inclusion criteria.
Blinding	Blinding was not relevant as all processing methods were done through available software with consistent parameters utilized across all treatment groups.

Reporting for specific materials, systems and methods

We require information from authors about some types of materials, experimental systems and methods used in many studies. Here, indicate whether each material, system or method listed is relevant to your study. If you are not sure if a list item applies to your research, read the appropriate section before selecting a response.

Materials & experimental systems

n/a	Involved in the study	
	x	Antibodies
	x	Eukaryotic cell lines
x		Palaeontology and archaeology
	x	Animals and other organisms
	x	Human research participants
x		Clinical data
x		Dual use research of concern

Methods

n/a	Involved in the study	
x		ChIP-seq
	x	Flow cytometry
x		MRI-based neuroimaging

Antibodies

Antibodies used

Antibodies for IHC
anti-Ki67 antibody (DAKO, MIB-1, 1:200)
Antibodies for IF
anti - ZO1 antibody (Servicebio, GB111402,1:500)
anti - claudin-1 antibody(Servicebio,GB11032,1:500)
anti-claudin-2 antibody(Abcam,ab53032,1:500)
Antibodies for western-blot
anti-claudin-2 antibody(Cell Signaling Technology, CST#48120,1:1000)
anti-Bcl-2 antibody(Cell Signaling Technology, CST#3498,1:1000)
anti-ZO-1 antibody(Abcam,ab221547,1:1000)
anti-occludin antibody(Abcam, ab167161,1:1000)
anti-claudin-1 antibody(Abcam,ab180158, 1:1000)
anti-cleaved caspase-3 antibody(Cell Signaling Technology,CST#9661,1:1000)
anti-BAX antibody(Abcam,ab199677,1:1000)
anti-β-tubulin antibody(Abcam,ab6046,1:2000)
anti-β-actin antibody(Abcam, ab227387,1:2000)
anti-GAPDH antibody(Abcam,ab8245,1:2000)
Secondary antibodies
Goat Anti-Mouse IgG antibody (ZSGB-BIO, ZB-2305, 1:5000)

	Goat Anti-Rabbit IgG antibody (ZSGB-BIO, ZB-2301, 1:5000)
Validation	All the antibodies used in this study are commercially available and have been verified by the manufacturers according to the data on their websites.

Eukaryotic cell lines

Policy information about cell lines

Cell line source(s)	C1498 mouse Acute myeloid leukaemia cell line obtained from ATCC(ATCC TIB-49)
Authentication	The cell lines were not authenticated
Mycoplasma contamination	Cells were routinely tested for mycoplasma and they tested mycoplasma negative
Commonly misidentified lines (See ICLAC register)	No commonly misidentified cell lines were used in this study

Animals and other organisms

Policy information about studies involving animals; ARRIVE guidelines recommended for reporting animal research

Laboratory animals	Specific pathogen free (SPF) mice on a C57BL/6J background were purchased from the Laboratory Animal Center of Shandong University. Female, 6-8 weeks. Mice were maintained on a 12-h dark/light cycle at ambient temperature (72±2F) with controlled humidity (~45%).
Wild animals	The study did not involve wild animals
Field-collected samples	The study did not involve field-collected samples
Ethics oversight	Animal protocols were approved by the Animal Ethics Committee of Qilu Hospital, Shandong University.

Note that full information on the approval of the study protocol must also be provided in the manuscript.

Human research participants

Policy information about studies involving human research participants

Population characteristics	Median age at enrollment 49 years<br>Male/Female: 49%/51%<br>Extended data available in supplementary table 1.
Recruitment	The samples of AML patients were collected from newly diagnosed patients in Qilu Hospital of Shandong University, and the samples of healthy participants were collected from healthy people in the physical examination center of Qilu Hospital of Shandong University
Ethics oversight	Our study was approved by the Medical Ethical Committee of Qilu Hospital of Shandong University (KYLL-2018-137).

Note that full information on the approval of the study protocol must also be provided in the manuscript.

Flow Cytometry

Plots

Confirm that:

- [x] The axis labels state the marker and fluorochrome used (e.g. CD4-FITC).
- [x] The axis scales are clearly visible. Include numbers along axes only for bottom left plot of group (a 'group' is an analysis of identical markers).
- [x] All plots are contour plots with outliers or pseudocolor plots.
- [x] A numerical value for number of cells or percentage (with statistics) is provided.

Methodology

Sample preparation	For all flow cytometry assays, following treatment or post-treatment incubation, cells were washed 2x in PBS. A total of 5×10^5 cells were resuspended in 100 ul of PBS, and antibody was incubated for 40 minutes in the dark.
Instrument	Beckman Gallios
Software	Kaluza 2.1.1

Cell population abundance	Cells were run to achieve > 30,000 events in the gated cell population.
Gating strategy	Gating was performed based on identifying a distinct population in FSC vs SSC plots. We ensure that a figure exemplifying the gating strategy is provided in supplementary figure 11.

☒ Tick this box to confirm that a figure exemplifying the gating strategy is provided in the Supplementary Information.
